# Eradication of HIV-1 latent reservoirs through therapeutic vaccination

**DOI:** 10.1186/s12981-017-0177-4

**Published:** 2017-09-12

**Authors:** Joshua Pankrac, Katja Klein, Jamie F. S. Mann

**Affiliations:** Department of Microbiology and Immunology, University of Western Ontario, London, ON N6A 5C1 Canada

**Keywords:** HIV-1, Latency, Cure, VLPs, CD4 T cells

## Abstract

Despite the significant success of combination anti-retroviral therapy to reduce HIV viremia and save lives, HIV-1 infection remains a lifelong infection that must be appropriately managed. Advances in the understanding of the HIV infection process and insights from vaccine development in other biomedical fields such as cancer, imaging, and genetic engineering have fueled rapid advancements in HIV cure research. In the last few years, several studies have focused on the development of “Kick and Kill” therapies to reverse HIV latency and kick start viral translational activity. This has been done with the aim that concomitant anti-retroviral treatment and the elicited immune responses will prevent *de novo* infections while eradicating productively infected cells. In this review, we describe our perspective on HIV cure and the new approaches we are undertaking to eradicate the established pro-viral reservoir.

## Background

As of 2015, the estimated global burden of HIV infection stood at 36.7 million individuals, with 2.1 million new infections and 1.1 million deaths from AIDS-related illnesses in that year alone. While 17 million people are now accessing anti-retroviral therapy (ART), in order to meet the Joint United Nations Programme on HIV/AIDS (UNAIDS) global target of “90-90-90” by the year 2020, another 15 million HIV-positive individuals must initiate and maintain ART. While ART has proven remarkably successful in transforming the HIV epidemic from a death sentence to a life-long, manageable illness, it remains non-curative, owing to its ability to target only actively replicating virus. This, coupled with the absence of an efficacious preventative vaccine, the expense of lifelong ART treatment to both the afflicted individual and government health care systems, along with the emergence of drug-resistant viral strains, necessitates that a curative approach becomes a major health priority.

## HIV infection and latent reservoir establishment

HIV is a sexually transmitted infection (STI) which is predominantly transmitted through the vaginal, anal, rectal and penile/foreskin mucosa. While the exact transmission process remains debatable, theories include: paracellular crossing of the mucosal barrier, capture by inter-digiting dendritic cells, transcytosis, and/or penetration through micro-fissures in the epithelium. Regardless, infectious foci are established within submucosal CD4 T cells minutes to hours following primary exposure. Within hours to days, HIV then establishes a systemic infection by reaching the local draining lymph node and disseminating around the body. Throughout this process, HIV is thought to undergo a series of genetic bottlenecks, such that a single infectious virion establishes an infectious event leading to viremia in most cases [1]. During the eclipse phase of viremia (~10 days), viral loads remain undetectable by conventional diagnostic techniques. This phase is proceeded by a detectable, rapidly amplifying acute infection, culminating with peak viremia and a concomitant collapse in CD4 T cell numbers. Heightened cytotoxic T lymphocyte (CTL) activity coincides with viral load decline, leading to the viral set point. The magnitude of the viral set point is a good diagnostic indicator of progression to Acquired Immune Deficiency Syndrome (AIDS). While the introduction of combination ART (cART) had a profound effect on the HIV landscape, infection remains non-curative. This is due to HIV’s ability to rapidly establish a transcriptionally silent reservoir within infected individuals. Evidence from non-human primate (NHP) studies suggests that the latent reservoir establishes within 3 days of infection, and that early detection and treatment might be insufficient to prevent latency establishment [2]. However, promising data indicates that early treatment with cART can reduce the reservoir size and may facilitate a “functional cure”, where viremia is controlled by yet unknown immune processes [3].

While numerous cell types are implicated as HIV proviral reservoirs, evidence suggests that the predominant cell type harboring inducible provirus are long-lived central memory CD4+ T cells. During acute infection, a robust adaptive immune response is activated with extensive proliferation of HIV-specific T and B cells. While anti-HIV CD4 T cells are instrumental to the immune response, they are also the primary targets of infection. The introduction of cART enables pharmacological control of ongoing viral replication by inhibiting various aspects of the viral replication cycle, thus resolving the activated immune response. During this contraction phase, activated HIV-specific CD4 T cells return to physiological levels by either transitioning into resting memory CD4 T cells or by dying through apoptosis. This transition may facilitate reservoir establishment due to the cells’ conversion to a lower metabolic state with fewer transcription factors such as NF-kB, NFAT and SP1. Thus, HIV latency could be due to the normal physiology of CD4 T cells transitioning between different cell stages and as an accidental side effect associated with cART treatment. However, this theory has since been challenged by reports suggesting that HIV latency is a virally encoded phenomenon that can be controlled by Tat protein, regardless of cellular activation [4]. It is because of HIV latency that cART is a life-long treatment whose interruption leads to rapid viral recrudescence. Overall, the establishment of a latent proviral reservoir, capable of rebounding viremia has become the major barrier to HIV cure.

## Eradicating the HIV reservoir

Numerous strategies have been evaluated to address the issue of HIV cure. The most promising tactic to-date involves a “Shock and Kill” approach. This method utilizes a pharmacological agent to ‘shock’ inducible and infectious HIV in the reservoir into transcriptional activity, thereby enabling its detection and elimination by immune or therapeutic mechanisms. Research has demonstrated that most HIV proviruses become integrated within the introns of actively transcribed host genes, and that the main HIV latent reservoir is found within resting memory CD4 T cells. Therefore, CD4 T cells are often the targets for latency reversal agents (LRAs), seeking to purge latent virus. Until recently, the most promising LRAs were the non-specific Histone Deacetylase inhibitors (HDACi), due to their in vitro ability to promote histone acetylation of integrated proviral promoters [5]. Prominent LRAs such as Vorinostat, Disulfiram, and Romidepsin have been tested in clinical studies as candidate LRAs to purge the HIV-1 reservoir [6–10]. Unfortunately, none could significantly impact upon the size of the reservoir, regardless of the promising preclinical research using both primary cells and cell lines [11]. More recently, in vitro latency reversal studies using two-drug regimens, incorporating HDACi and protein kinase C (PKC) agonists, have been shown to synergistically amplify latency reversal, providing support that an effective “shock” is achievable. Although HDACi were once highly promising, several alternative studies have reported minimal latent HIV-1 reactivation in primary CD4+ T cells ex vivo [12]. Additionally, evidence suggests that certain HDACis may suppress immune responses through inhibited cytokine release, delayed killing of activated infected-CD4+ T cells, impaired CTL functioning, and unwanted apoptosis of NK cells [13]. Furthermore, certain HDACis have immunomodulatory effects on B cells and inhibit primary germinal center responses [14]. Finally, HDACi are non-specific T cell activators, which could theoretically cause the propagation of infected cells. New and improved LRAs are necessary to facilitate reservoir eradication and cure.

As disseminating HIV infection results in recruitment and activation of CD4 and CD8 T cells, it is thought that the anti-viral T cell response, although able to exert some level of viremic control, also fuels the HIV infection. Consequently, during acute infection, excluding any confounding STIs that may activate immune responses, it can be rationalized that anti-HIV CD4 T cell responses would be enriched more than T cell receptors (TCRs) with alternative antigen specificities, thus becoming candidate targets for latency establishment. Findings from our group and others suggest that most latently infected cells within the blood compartment of HIV-infected individuals appear to express TCRs specific for HIV peptides but not to control antigens, including PPD and Flu/Tetanus/CMV cocktails [15–17]. Furthermore, non-specific TCR activators such as CD3/CD28 will result in de novo viral RNA production in HIV-infected CD4 T cells [18, 19]. Collectively, these implicates T cell activation pathways as potentially important to achieving latency reversal. Furthermore, antigen presenting cells (APCs), such as dendritic cells (DCs), were shown to induce contact-dependent latency reversal [20, 21]. Taken together, we hypothesized that if the largest pool of latently infected cells are HIV-specific resting memory T cells, they might be more efficiently activated and purged using a highly polyvalent vaccine preparation that is representative of the near-complete viral quasispecies. A highly representative vaccine is more likely to have its proteins processed and presented to latently infected T cells bearing HIV-specific TCRs. As proof of principle, we have conducted latency reversal studies in HIV-infected peripheral blood mononuclear cells (PBMCs) derived from infected volunteers that were treated during acute stage infection. We have constructed a polyvalent virus-like particle (VLP) formulation from HIV RNA isolated from infected individuals and for use as our latency reversing activating vector (ACT-VEC). Preliminary data suggests that ACT-VEC, when used to pulse DCs co-cultured with HIV-infected CD4 T cells, causes significant transcription of HIV RNA. Furthermore, ACT-VEC-induced latency reversal exceeds that of promising 1-drug and 2-drug LRA regimens presently in clinical trials. Ongoing studies evaluating ACT-VEC-mediated latency reversal in CD4 T cell cultures derived from chronic and pediatric volunteers will help characterize our formulation’s efficacy at different stages of disease and immune development. The pediatric cohort is particularly interesting as findings in the “Mississippi Child”, a perinatally HIV-infected child that was placed on ART shortly after birth, revealed a detectable decay in reservoir size following ART cessation. Logic suggests that underdeveloped immunological memory and immunoregulation are possible factors limiting reservoir size in this instance. Future studies on potential delivery and adjuvant strategies for ACT-VEC will enable us to understand whether ACT-VEC could function as an immunological priming strategy to facilitate the “Kill” component of the therapy [22]. Of note, in the SIV-NHP model of infection, T cell vaccines delivering SIV proteins can elicit cellular immune responses, reduce viral loads, and preserve memory CD4 T cell numbers [23–25]. As ACT-VEC represents the entire proteome and morphology of wildtype virus, it is plausible it could elicit similar humoral and cellular immune responses.

## Conclusion

While there is clearly some ways to go in the development of ACT-VEC as a clinically relevant pharmacologic, it represents a promising candidate for “Shock and Kill” treatment. Just as major efforts were successfully invested into developing effective ART to combat the HIV associated death sentence, significant efforts and capital have also been invested into the creation of a prophylactic vaccine. Accordingly, the prospect of an HIV cure, once thought to be impossible, has now become a plausible scientific goal and demands the utmost priority. This is especially relevant now that one individual, the “Berlin Patient” is cured of HIV infection, and many individuals within the “VISCONTI” cohort appear to be functionally cured, i.e. controlling their HIV-infections for several years upon cART cessation [26]. Whether the cure that is eventually realized is functional or sterilizing in nature remains to be seen. However, such ambitious goals require additional emphasis on HIV research, and greater support for funding agencies and scientists by both government and private organizations.
